# Protecting Migration Corridors: Challenges and Optimism for Mongolian Saiga

**DOI:** 10.1371/journal.pbio.0060165

**Published:** 2008-07-29

**Authors:** Joel Berger, Julie K Young, Kim Murray Berger

## Abstract

Hunting pressure and habitat loss place the endangered saiga, a type of antelope that was once abundant in central Asia, at high risk of extinction, and make the protection of the migratory routes of Mongolian populations even more critical for conserving the species.

Migrations are an important ecological phenomena rapidly declining throughout the world [1]. Within many ungulate populations, migration is a polymorphic trait; animals can cover either long or short distances, pass across broad swaths of land such as those of caribou (Rangifer tarandus) and wildebeest (Connochaetes taurinus), or squeeze through bottlenecks as narrow as 120 meters as described for pronghorn (Antilocapra americana) [2,3]. Given that the persistence of terrestrial migration is challenged primarily by anthropogenic forces, protection is often possible, assuming the availability of appropriate knowledge concerning movements, threats, and meta-population structure, and the willingness to implement coincident conservation actions that involve local decision makers. Here, we illustrate these issues by profiling an endangered species—the Mongolian saiga (Saiga tatarica mongolica; Figure 1), highlighting the importance of protecting movement routes in light of habitat, human culture, and other sources of population risk.

**Figure 1:**
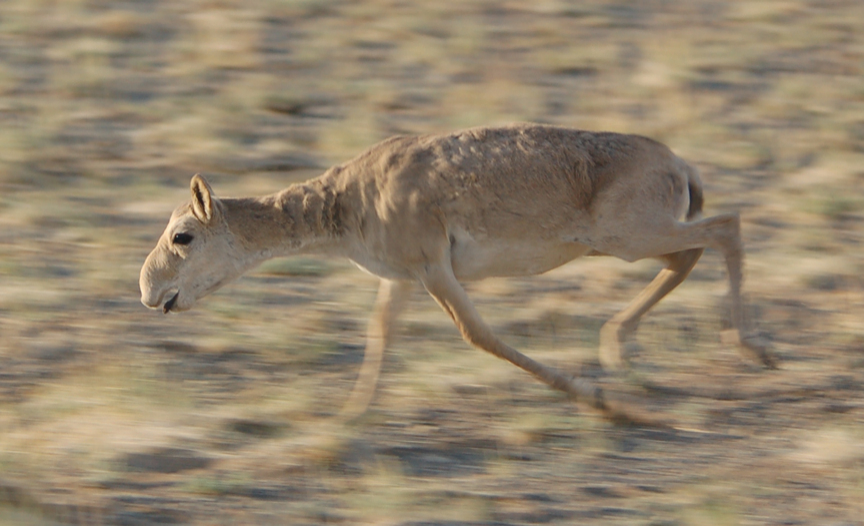
Mongolian Saiga (Saiga tatarica mongolica) Juvenile Mongolian saiga female within the Sharga Nature Reserve. Over-harvesting, poaching for horns, and habitat degradation are among the threats facing this endangered migratory species.

## Mongolian Saiga

Expansive grasslands in central Asia sustain extraordinary movements between winter and summer ranges of several endangered species [4,5]. Many of these wide-ranging species are threatened or endangered because of over-harvesting, poaching for horns that are subsequently used in traditional medicine, and degradation of pastures by livestock grazing [6]. Saiga are among the unfortunate leaders in terms of population declines, their numbers having dropped more than 95%, from greater than 1,000,000 to less than 50,000 in under two decades [7]. Only two subpopulations remain within Mongolia, totaling approximately 5,000 individuals [7]. Recent anti-poaching efforts may halt these downward trends, but protection from threats other than poaching has been hampered by a lack of knowledge about movements and locations at which to focus conservation efforts. Thus, information on migration routes and potential impediments to movement may reduce the loss of corridors and facilitate saiga persistence before saiga populations reach perilously low numbers.

## Importance of Connectivity for Saiga Populations to Persist

**Figure 2:**
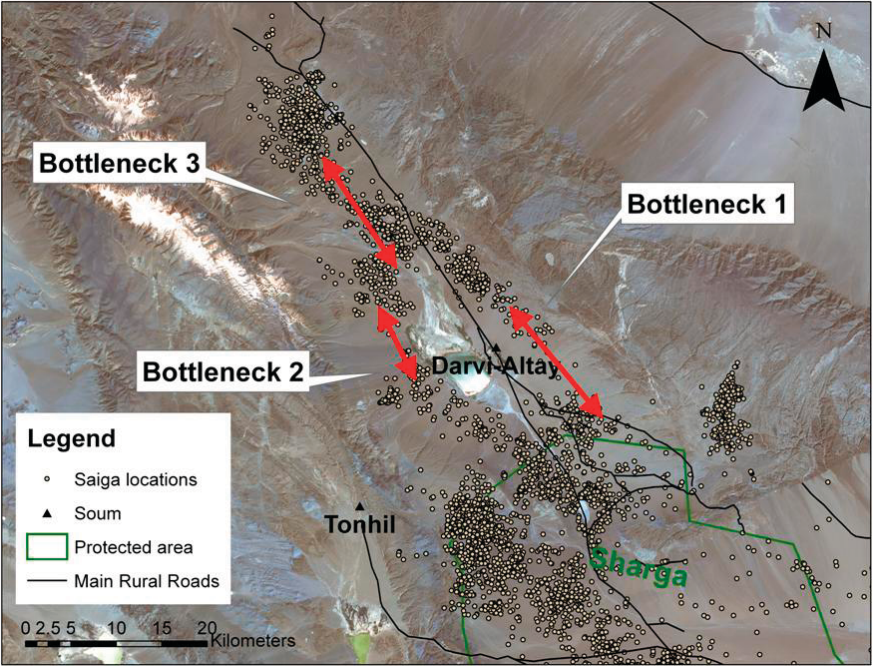
Potential Bottlenecks along Saiga Migratory Corridors Map of northwestern section of Sharga Nature Reserve, showing nearby soums (towns), roads, and three restricted movement zones outside of the reserve (1: skirting Darvi-Altay soum; 2: passing Darvi Lake; 3: valley less than five kilometers wide connecting Shargyn Govi with Huysiyn Govi). Movements are based on locations of GPS-collared adult female saiga captured within the Reserve boundaries [8].

Our research on adult female saiga using global positioning system (GPS) collars identified the use of a narrow corridor connecting the two subpopulations north of the Altai Mountains in western Mongolia [8]. Females that used the Sharga Nature Reserve moved beyond reserve boundaries at least twice within the 2006–2007 monitoring period, traveling northward into currently unprotected areas that connect the Shargyn Govi and Huysiyn Govi subpopulations. The corridor for saiga moving between the Shargyn Govi and the Huysiyn Govi includes three potential bottlenecks: a passage around Darvi-Altay soum (town), a second along the lake north of Darvi-Altay soum, and a third area less than five kilometers wide located north of the lake and Darvi-Altay (Figure 2). Although all three bottlenecks likely represent important passages, the first two serve as alternative routes; thus the loss of either one is unlikely to significantly impact saiga movement. The third potential bottleneck, however, represents the only known route through that area [8]. Therefore, the disruption or cessation of this corridor could lead to population collapse, either by eliminating opportunities to migrate to avoid inclement weather or by assuring the collapse of meta-population structure.

The narrowness of these three locations is, in and of itself, not a cause for biological concern; pronghorn migrate through multiple narrow bottlenecks along their longest migratory route south of the Canadian border [3]. However, the apparent bottlenecks used by saiga also include the location of relatively permanent features (i.e., soum and lake) in addition to their proximity to the primary road that connects Darvi-Altay to other soums (Figure 2). Saiga movement within these areas is further restricted because all three potential bottlenecks occur within an area grazed extensively by domestic goats and sheep, which directly compete with Mongolia's wildlife for forage [9]. The mountainous topography confines saiga movements within these corridors, and because of heightened livestock use and close proximity, there exists potential risk of disease transmission with detrimental effects to both saiga and livestock [10]. While these data provide important insights on apparent bottlenecks that may represent areas more vulnerable to threats, the imminence of such threats is still unclear within this system. Nevertheless, if saiga meta-population structure is to persist, saiga movement must be maintained despite the burgeoning populations of humans and livestock and increasing vehicle traffic.

## Corridor Conservation Recommendations

By gathering baseline information on potential impediments to movements, an opportunity now exists to engage the local community in an important dialogue about how to maintain connectivity for saiga in the face of increasing development and grazing pressure. The Sharga Nature Reserve contains a mixture of wildlife, people, and ever-increasing numbers of livestock. No signs demarcate the boundaries, and the reserve has neither staffing nor infrastructure. The fact that saiga persist within the reserve is likely by default—Mongolia is one of the least densely populated countries in the world, and the nomadic lifestyle of its people precludes the ex-urban development and fence lines rapidly enveloping other developing nations [11]. A nomadic society, like that in the Mongolian saiga's range, typically utilizes rangelands in ways that are beneficial to both livestock and endemic wildlife [12]. However, the sociopolitical changes that occurred within Mongolia over the past few decades have created new economic opportunities for both nomadic pastoralists and for the expansion of soums [13]. It is likely that Sharga Nature Reserve and surrounding areas will be faced with increased vehicle traffic along with the expanding human population, rising per capita wealth, which leads to an increase in personally owned vehicles, and expanding global interest in eco-tourism to remote regions of the world. Further, plans to pave a major road linking western Mongolia with the Chinese border will likely result in increased traffic within this region. The impact of these potential threats is relatively unknown. Thus, garnering information on saiga movements and meta-population structure is a necessary step, but not sufficient to assure long-term conservation.

The success of protecting this corridor, and many like it globally, hinges on approaches that involve local people in consort with government agencies. In the case of Mongolian saiga, the human side includes local herder communities and government. Discussions currently focus on expanding the boundaries of the Sharga Nature Reserve to protect the corridor. Although this is an important first step, such an expansion is likely to have little impact on saiga numbers if it is in name alone. Current protected areas are little more than paper parks, and anti-poaching rangers often lack necessary field equipment, transportation, and statutory authority to conduct their jobs [14].

Similar threats plague migratory wildlife worldwide, sometimes spanning international boundaries. Our best hope to protect critical corridors is through open dialogue with the local communities, land managers, and government officials before crisis situations occur. The scientific community has an important role to play by providing data to identify potential threats. Ultimately, however, it is only through dialogue with vested interests that recommendations to reduce threats can be implemented. Protecting corridors will necessitate addressing difficult issues, but baseline data provide opportunities to engage in these discussions before situations become dire.
